# Editorial

**Published:** 1951-07

**Authors:** 


### Editorial

**Our Function**

Nearly twenty years ago* the functions of the Provincial Medical Journal were thus described: "It gives opportunities for recording cases that would be crowded out of the larger general medical publications, or would be unacceptable to the specialized periodicals. It stimulates medical men of the district to send much that might otherwise be lost: and to prepare their communications with greater precision, since they will afterwards be published. Finally, a local journal forms a mirror of the activities of the medical men of the district, it records passing events. . . ."

As to the last, we can only record those events of which we are cognisant: and are very much dependent upon local correspondents and the secretaries of various medical societies, having in fact a constant fear of our shortcomings in this respect. In recording the proceedings at local meetings, we are much at the mercy of authors. It is manifestly absurd for a quarterly journal to reprint a communication already published several weeks before.

As far as concerns the Bristol Medico-Chirurgical Society, the *Journal* has the RIGHT to publish the proceedings†. But there is a growing tendency for those who address the Society to ignore that right: and, curiously, this is specially noticeable in the case of Distinguished Visitors, the very persons whose addresses we most wish to place on record. It may be, of course, at times that the subject is not really suitable for discussion at a meeting attended mainly by general practitioners. Perchance it is something specially important, deserving a wider publicity than the "Proceedings" of an obscure provincial society: or is reserved for further use on some other occasion. But of late years only too often the impression remains that the Distinguished Visitor just couldn't take the trouble to think out beforehand what he was going to say, far less to write it down! Maybe members are so flattered that these exalted personages deign to address them that they are ready to ignore deficiencies

* Nixon, J. A.—"Provincial Medical Journals", Hist. of Med. Sectn., B.M.A., London, 1932. *Bristol Med. Chir. Journ.*, XLIX, p. 318.

† Unless specially excepted under Rule 19.

—even of manners. If not, it might be well to remind *all* who address the Society that we expect their communications to be available for publication.

*Ita vita est hominum quasi quum ludas tesseris.*
*Si illud quod est maxume opus jactu non cadit,*
*Illud quod cecidit forte, id arte ut corrigas.*
*Terent.*

**Old Age**

Politicians may deplore " an ageing population " as if it were caused by a wicked conspiracy to frustrate " Planning ". Actually, of course, it means only that many more now live out their full span: there are, in this island, above three times as many people over sixty-five as there were fifty years ago. In this we may well feel some professional pride. But pleasing though it is, this has in turn produced problems which we have not yet solved. For example, there is the very greatly increased need for hospital accommodation for these old people: much of our last issue was devoted to methods of using what we have to the best advantage.

But many of the problems are social and political rather than medical. With strictly limited resources, are we to give preference to the child, whose whole life may be changed by timely treatment; to the family breadwinner; or to the old man who has earned care and comfort by a life of toil? These are questions for all alike: certainly not for the medical profession alone. Let Shaw sneer, this is not the *doctor's* dilemma. Premier, poet, prostitute, paranoiac, to the priest each is alike and equally a penitent, to the physician a patient. (Only a politician could have thought out " Positive Health ": the practitioner knows well that his main business is with the care of the sick.*) Pressure on housing may well mean that if the elderly patient enters hospital for necessary treatment, he will lose the one room he can call his own, and his few bits of furniture—and then what happens when he is fit to leave hospital again? Again, the Welfare State seems to be destroying all family and social responsibility: " Oh, NO, doctor! *We* can't look after father now he needs nursing".

There is some evidence that our masters begin to understand that the population changes mean all must work harder or longer. Consultants and civil servants may now work until sixty-five, magnates and magistrates until seventy. Perhaps we

* Luke V, 31.

shall soon be allowed to continue, like Cabinet Ministers, as long as we seem able. It is not only that for many the "Pension" implies a cruel drop in living standards; " Retirement " is, by itself a very deadly disease.* " The first cause, which no man living can avoid, is Old Age: which being of the same quality as melancholy is, must needs cause it. And common experience confirms the truth of it in old persons, especially such as have lived in action all their lives, had great employment, much business and many servants, and leave off *ex abrupto*: they are overcome with melancholy in an instant."† Only a few have had great employment: but many humbler people, who have led busy and (they trust) useful lives, are just as profoundly affected. Others again, for example, have been keen and regular supporters of club or lodge, perhaps have long carried the tedious and obscure burdens of junior office—gradually as the years pass the realisation grows that the expected reward, President, Grand Master, Great Sachem, will never come. Instead, there creeps in the fear of becoming ὁ κατέχων.‡ The tragedies of age are not entirely financial or physical.

* "Among factors influential in causing people to commit suicide are . . . the relation between our present society and old people. . . . Suicide in the last fifty years has increasingly become a disorder of elderly people ". Swinscow, D., Some Suicide Statistics; *B.M.J.*, 1951, i, 1417.

† *Anatomy of Melancholy*, Democritus Junior; I, § 2, ii (5).

‡ " He who now letteth will let until he be taken away." II Thessal. II. 7.

